# Mitochondrial Research: Yeast and Human Cells as Models

**DOI:** 10.3390/ijms23126654

**Published:** 2022-06-15

**Authors:** Maša Ždralević, Sergio Giannattasio

**Affiliations:** 1 Faculty of Medicine, University of Montenegro, Kruševac bb, 81000 Podgorica, Montenegro; masaz@ucg.ac.me; 2 Institute of Biomembranes, Bioenergetics and Molecular Biotechnologies, National Research Council, 70126 Bari, Italy

The evolution of complex eukaryotes would have been impossible without mitochondria, key cell organelles responsible for the oxidative metabolism of sugars and the bulk of ATP production. Mitochondrial enzymes are central to amino acid, nucleotide and fatty acid metabolism, which also generate biosynthetic intermediates, providing cells with reducing power and metabolic energy. A unique feature of mitochondria is that they contain their own genome as well as biosynthetic machinery to produce RNA and proteins. The importance of mitochondria for cell physiology surpasses by far their role in energy production, as it is now well established that mitochondria are involved in Ca^2+^ homeostasis, regulation of apoptosis, activation of endoplasmatic reticulum (ER) stress response and in general, in receiving, integrating and relaying intracellular signals. Mitochondria form highly dynamic networks in the cell; processes of fission and fusion allow cells to adapt to changeable energetic demands, as well as to counteract damage. This adaptive response to nutritional and environmental cues is under tight nuclear control and is achieved by coordinate gene expression regulation through so-called anterograde signalling. If the stressors are harsh enough to provoke mitochondrial dysfunction, damaged mitochondria respond by activating the retrograde signalling pathway, which changes nuclear gene transcription patterns in order to counteract the stress and maintain cell homeostasis. With the discovery of pathogenic mitochondrial DNA defects in the 1980s, mitochondrial dysfunction is now recognized as a common factor underlying many pathological conditions.

Recognition of their vital role in many different physiological and pathophysiological processes and in the maintenance of eukaryotic cell homeostasis is responsible for the continuous growth of the mitochondrial research field. This Special Issue, entitled: “Mitochondrial research: yeast and human cells as models”, gathers the most recent contributions to the understanding of mitochondrial function and regulation at the leading edge of cell biology research.

Unique characteristics of the physiology of *S. cerevisiae*, such as its nature of facultative aerobe as well as its ability to survive severe mitochondrial dysfunction, made it a very attractive model to study mitochondrial involvement in different cellular processes. As very nicely reviewed by Rouchidane Eyitayo et al. many different powerful tools of yeast molecular genetics, such as ectopic expression of mammalian homologs, were used to investigate the role of mitochondria in the apoptotic process [1]. In particular, they focused on the role of subcellular localization of BCL-2 family members as major regulators of apoptotic cell death in mammals, and showed that it is closely related to the dynamics of the intracellular membranes. The role of intrinsic molecular determinants, as well as mitochondrial receptors, mitochondria-associated membranes and mitochondria-ER contacts (MERC) in pro-apoptotic BAX and anti-apoptotic BCL-xL dynamics were discussed. A better understanding of the regulation of BCL-2 protein localization is of particular interest in the optimization of anti-cancer therapies since BAX is one of the targets of serine/threonine kinase (AKT) and is constitutively active in many cancers [2]. 

Koushi and Asakai reported on the role of the ATP synthase and ATP/ADP carrier (AAC) in formation of the permeability transition pore (PTP) in yeast *S. cerevisiae* [3]. By using bisindolylpyrrole (BP), an activator of the PTP, they showed that BP-induced mitochondrial depolarization was sensitive to cyclosporine A (CsA), an inhibitor of PTP, and CsA-sensitive proline rotamase (Cpr3), a CsA-inhibitable chaperone, mediated by AAC. This CsA-dependent depolarization of mitochondrial membrane potential could be used for the screening of small molecules as potential PTP modulators.

Mitochondrial dysfunction is known to be linked with the onset of neurodegenerative diseases, cancer and diabetes [4]; therefore, cells have deployed quality control mechanisms at multiple levels in order to maintain mitochondrial functionality. Kumar and Reichert highlighted the role of mitophagy, a selective autophagy of damaged and/or dysfunctional mitochondria, in different physiological or stress conditions [5]. Molecular determinants and mechanisms of yeast (*S. cerevisiae*) mitophagy are discussed, focusing on the role of Atg32, an essential mitochondrial receptor for mitophagy. In addition, the role of mitochondrial fission in promoting mitophagy is emphasized. Finally, signalling pathways that regulate both ubiquitin-dependent and -independent mitophagy in yeast and mammals are discussed. A better understanding of these mechanisms has the potential to contribute to the therapeutic treatment of neurodegenerative and other disorders with mitochondrial involvement by impacting mitophagy pathways.

In general, mitochondrial diseases do not have effective therapeutic modalities to date, and as presented by di Punzio et al. yeast *S. cerevisiae* yet again proved its potential as an excellent model organism, this time in drug screening [6]. More precisely, yeast-based screening was used to find new active molecules against mitochondrial diseases associated with dominant mutations in *ANT1*, the gene encoding one of the subunits of the ADP/ATP carrier. *ANT1* mutations are found in different degenerative mitochondrial pathologies, and this study identified five molecules with potential therapeutic benefit. The value of the yeast model is particularly emphasized in a context such as this, where no adequate mammalian model exists. 

Mutations affecting mitochondrial translation machinery are known as one of the main causes of mitochondrial pathologies. In particular, Figuccia et al. reported on the role of mitochondrial aminoacyl-tRNA synthetase (ARS) variants identified by next-generation sequencing (NGS) in different disorders [7]. Since mtARSs are conserved through evolution, it has been possible to use yeast as a model for functional studies of mt*ARS* gene mutations. Although being a single-cell microorganism poses limitations on the use of yeast in evaluation of pathogenicity of miARS variants on the tissue and/or organ level, its effects on intracellular metabolism are well studied. The advantages of yeast molecular genetics are made evident in this example as well, since in yeast it is possible to study the effects of each single variant with an identical genetic background, which is impossible to do in mammals. Moreover, in exceptional cases when mt*ARS* pathological mutations are found, for example, in genes that encode both mitochondrial and cytoplasmic isoforms, in yeast it is possible to study the effect of mutation specifically on mitochondrial function. 

The role of mitochondria in desiccation tolerance has already been described, but Chen et al. reported on the very important role of mitochondrial dynamics in this process [8]. They demonstrated that preserved mitochondrial dynamics during the stationary phase of growth is necessary for adequate cell response to dehydration/rehydration stress. This was correlated to the maintenance of the mitochondrial genome as well, since mtDNA loss is associated with lower desiccation tolerance.

Changes in mitochondrial functionality trigger a mechanism known as “mitochondrial retrograde signalling”, by which cells respond and adapt to said mitochondrial dysfunction by extensive changes in nuclear gene expression [9,10]. Retrograde (RTG) signalling is known to be involved in the development and differentiation of yeast colonies, as reported by the group of Palková [11], which further analysed proteomes of cell subpopulations: U cells and L cells in the upper and lower colony regions, respectively [12]. Rtg proteins were shown to regulate different metabolic processes, predominantly in U cells, which is important in their adaptation to changes in nutritional conditions. In addition, regulation by Rtg factors was shown to be of particular importance for mitochondrial function in colonies. 

The research articles in this Special Issue witness the relevance of yeast as a model organism for 21st century biology [13] and its complementarity with human cell models. The use of yeast as a model has allowed for new achievements in the research on the multiple pathways of crosstalk between mitochondria and other cell organelles and components, thus laying the basis for the elucidation of the mitochondrial role in health and disease.

## Data Availability

Not applicable.
